# From Microspikes to Stress Fibers: Actin Remodeling in Breast Acini Drives Myosin II-Mediated Basement Membrane Invasion

**DOI:** 10.3390/cells10081979

**Published:** 2021-08-04

**Authors:** Julian Eschenbruch, Georg Dreissen, Ronald Springer, Jens Konrad, Rudolf Merkel, Bernd Hoffmann, Erik Noetzel

**Affiliations:** Institute of Biological Information Processing 2 (IBI-2): Mechanobiology, Forschungszentrum Jülich, 52428 Jülich, Germany; j.eschenbruch@fz-juelich.de (J.E.); g.dreissen@fz-juelich.de (G.D.); r.springer@fz-juelich.de (R.S.); j.konrad@fz-juelich.de (J.K.); r.merkel@fz-juelich.de (R.M.); b.hoffmann@fz-juelich.de (B.H.)

**Keywords:** filopodia, invadopodia, focal adhesion, basement membrane disruption, breast cancer invasion, actomyosin, cell force, mechanical probing, epithelial mechanobiology

## Abstract

The cellular mechanisms of basement membrane (BM) invasion remain poorly understood. We investigated the invasion-promoting mechanisms of actin cytoskeleton reorganization in BM-covered MCF10A breast acini. High-resolution confocal microscopy has characterized actin cell protrusion formation and function in response to tumor-resembling ECM stiffness and soluble EGF stimulation. Traction force microscopy quantified the mechanical BM stresses that invasion-triggered acini exerted on the BM–ECM interface. We demonstrate that acini use non-proteolytic actin microspikes as functional precursors of elongated protrusions to initiate BM penetration and ECM probing. Further, these microspikes mechanically widened the collagen IV pores to anchor within the BM scaffold via force-transmitting focal adhesions. Pre-invasive basal cells located at the BM–ECM interface exhibited predominantly cortical actin networks and actin microspikes. In response to pro-invasive conditions, these microspikes accumulated and converted subsequently into highly contractile stress fibers. The phenotypical switch to stress fiber cells matched spatiotemporally with emerging high BM stresses that were driven by actomyosin II contractility. The activation of proteolytic invadopodia with MT1-MMP occurred at later BM invasion stages and only in cells already disseminating into the ECM. Our study demonstrates that BM pore-widening filopodia bridge mechanical ECM probing function and contractility-driven BM weakening. Finally, these EMT-related cytoskeletal adaptations are critical mechanisms inducing the invasive transition of benign breast acini.

## 1. Introduction

Cell migration through basement membranes (BM) is a crucial step in cancer progression and invasion [1]. Invasive cells degrade the dense sheet-like collagen scaffold of the BM by matrix-metalloproteases (MMP). In addition, cellular forces drive local BM disruption [2,3]. Matured BM scaffolds act as physical barriers capable of sustaining physiological mechanical loads in homeostatic microenvironments [4,5]. In cancer progression, the tumorous extracellular matrix (ECM), characterized by oncogenic EGFR signaling and increased local stiffness, induces cell-force generation, finally fueling BM disruption [6]. Invasive cells undergo epithelial to mesenchymal transitions (EMT) programs with a fundamental reorganization of actin cytoskeletal structures. EMT cells form excessive sensory projections, spike-like filopodia, and actin-rich invadopodia. Moreover, enhanced cell contractility by actin stress fiber formation facilitates cell spreading and migration through a confining ECM [7]. EMT and pro-invasive protrusive activity are, among other things, induced by transforming growth factor-β (TGFβ) and epidermal growth factor (EGF) signaling pathways [8,9]. Actin-rich cell protrusions (CP) play a crucial role in cell migration and ECM mechanosensing. Their mechanical and sensing activity depends on ECM anchorage via stable matrix focal adhesions (FA) [10]. Force-transmitting FAs connect the cell cytoskeleton with the ECM via myosin II-coupled actin stress fibers (SF). The presence of myosin II is essential for actin filament contraction [11].

CPs are classified as lamellopodia, pseudopodia, podosomes, filopodia or filopodia-like protrusions, and invadopodia. This categorization is based on shape, the presence and distribution of actin scaffolding proteins, proteolytic enzymes, and cell type [12,13]. Invadopodia formation has been found in vivo and human cancer cell lines [14,15]. Albeitstrongly associated with invasive cells, invadopodia are heterogeneous in form and function. They often contain membrane-tethered proteases such as MT1-MMP (MMP14) and actin-bundling factors such as Tks5 and cortactin [16,17,18]. They also exert mechanical forces on the surrounding ECM, facilitating local matrix degradation and cell migration [19]. Additionally, invadopodia formation is induced by increased intracellular tension, matrix rigidity, contractile cell forces, and growth factor signaling [20,21]. Moreover, invasive cancer cells form force-transmitting filopodia or filopodia-like protrusions [12]. Enhanced density of filopodia-like actin spikes correlates with high invasiveness of ovarian cancer cells [22]. Studies on the mechanistic aspects of actin protrusion function in cancer invasion and exist for single cells migrating in 3D matrices [12,23,24,25]. In contrast, it is not yet understood how actin reorganization contributes to the invasion of epithelial cells through BM barriers. Herein, the direct cellular interaction with the BM scaffold and the underlying ECM remains elusive.

Therefore, we aimed at an understanding of the mechanisms of cellular mechanosensing across BM barriers and how these promote invasion. We focused on the functional interplay of actin CPs and actomyosin contractility as the initiating step of BM disruption. Our work characterized BM- and ECM-sensing actin CP in benign breast cell acini. We assessed such acini with a non-transformed genetic background to resemble invasive BM disruption mechanisms activated by changing ECM cues. We used tumor-like EGF and stiffness conditions to interlink CP regulation with invasive cell transition. In non-invasive acini, actin CPs originated from the cortical actin network in the lateral basal cell layer and interacted with the BM. In invasion-triggered acini, two cell populations were discovered at the BM–ECM interface. Their actin cytoskeletons were dominated either by mechanosensing microspikes (MS) or by contractile stress fibers (SF). Actin MS were reminiscent of the laterally formed CPs that lack invasion-related MT1-MMP. By combining high-resolution confocal microscopy and traction force microscopy (TFM), we mapped the MS to the SF transition process in response to tumor ECM conditions. Cells with initially high coverage of ECM-directed MS progressively reinforced their cytoskeleton by forming contractile SFs. Furthermore, we functionally link these SF-rich cells with hot spots of high actomyosin-generated force. Finally, our work suggests a functional link between actin cytoskeleton reorganization and SF-derived mechanical forces that weakens the BM at potential sites of cell invasion.

## 2. Materials and Methods

### 2.1. Cell Maintenance

MCF10A cells were purchased from ATCC (Manassas, VA, USA) and maintained in culture dishes under standard culture conditions (37 °C, 5% CO_2_) in DMEM/F12 growth medium (ThermoFisher Scientific, Waltham, MA, USA) containing 5% horse serum (ThermoFisher Scientific), 0.5 μg/mL hydrocortisone, 100 ng/mL cholera toxin, 20 ng/mL EGF, 10 μg/mL insulin (all Sigma Aldrich, St. Louis, MO, USA), 100 U/mL penicillin, and 100 μg/mL streptomycin (ThermoFisher Scientific). For MCF10A 3D morphogenesis, a DMEM/F12 assay medium was applied (see below). In addition, an MCF10A cell variant was transduced with RFP-LifeAct (IBIDI, Munich, Germany) to visualize the actin cytoskeleton in live-cell imaging experiments.

### 2.2. MCF10A Morphogenesis and Isolation from EHS Matrix

Single MCF10A cells were seeded on top of the growth-factor-reduced EHS substrate (Geltrex, ThermoFisher Scientific) to generate ld-BM (day 11) and hd-BM (day 21). Seeded cells were cultivated from day one to day nine in assay medium with 5 ng/mL EGF and from day 9 to day 21 in assay medium without EGF [4]. For further analyses, acini were isolated from EHS matrix and washed with ice-cold PBS and incubated in 2 mL ice-cold cell recovery solution (CRS) (BD Biosciences, San Jose, CA, USA) for 30 min (4 °C) to depolymerize the EHS matrix, as previously described [5]. Next, acini were washed with fresh EGF-free assay medium and individually picked under a stereo microscope for seeding onto 35 mm cell culture dishes, either with microscopic glass bottoms or elastomeric substrates. Acini adhered to Geltrex-coated substrates for 15 min (37 °C, 5% CO_2_) and were subsequently covered with 2 mL fresh EGF-free assay medium or growth medium (20 ng/mL EGF). The time point of cell seeding was defined as assay start.

### 2.3. Preparation and EHS-Protein Coating of Elastomeric Substrates

Acini were seeded on an 80-µm-thick layer of cross-linked PDMS silicone rubber substrates (Sylgard 184, Dow Corning, Midland, MI, USA) with an elasticity of 12 kPa (mixing ratio of base oil and cross-linker oil 50:1 per weight). The layer thickness of 70 µm was set by spin coating on 100 µm thin cover slips (Cover Slip, Ø22 mm, #0, Menzel-Gläser, Braunschweig, Germany). Silicone-coated cover slips were glued to the bottom of 3.5 cm Petri dishes to cover predrilled 1.8 cm holes [26]. Young’s modulus and Poisson’s ratio of elastomer samples were determined, as described previously [27]. Before cell and acini seeding, elastomeric substrates and glass substrates (Cover Slip, 24 × 24 mm, HP, Menzel-Gläser, Braunschweig, Germany) were functionalized for cell adhesion with 600 μL of non-gelling protein solution of Geltrex (20 μg/mL) in ice-cold PBS for 18 h at 4 °C.

### 2.4. Biochemical Treatments

Acini were transferred onto glass and elastomeric substrates and incubated 15 min after seeding either with EGF-free assay medium or with growth medium containing 20 ng/mL EGF [4] for defined periods of 1, 8, or 24 h. For inhibition of cellular myosin activity, blebbistatin was added directly after seeding and kept on cells for the entire experiment. Cells were treated with 25 µM blebbistatin (B0560 Sigma-Aldrich) and solved in dimethyl sulfoxide (DMSO) (final DMSO concentration = 0.3%).

### 2.5. Immunofluorescence (IF) Staining

Acini were fixed for 20 min with 3.7% paraformaldehyde in cytoskeleton-buffer (CB: 5 mM EGTA, 5 mM glucose, 10 mM MES, 5 mM MgCl_2_, 150 mM NaCl, 1 g/L streptomycin; all Sigma Aldrich), washed for 5 min with 20 mM glycine-CB (Sigma-Aldrich), and permeabilized with 1% Triton-X 100-CB (Sigma-Aldrich) at RT. Non-specific antibody binding was blocked with 5% skim milk powder (Sigma Aldrich) and 1% AffiniPure F(ab’)2 fragment goat anti-mouse IgG, 115-006-006, Jackson ImmunoResearch, West Grove, PA, USA) in CB for 2 h at RT. Acini were incubated overnight at 4 °C with the primary antibodies (anti-type IV collagen ab6586, Abcam, Cambridge, 624 England, UK; anti-cortactin (p80/85) clone 4F11, Merck, Darmstadt, Germany; anti-MMP14 (MT1-MMP) MAB12762 Abnova, Taipei City, Taiwan; anti-pMLCK 3671 Cell Signaling, Danvers, MA, USA; anti-talin clone 8d4, T3287 Sigma-Aldrich; anti-TKS5 (fish M-300) sc-30122 Santa Cruz Biotechnology, Dallas, TX, USA; anti-vinculin clone hVin-1, V9131 Sigma-Aldrich) diluted in 1% skim milk powder in CB (for pMLCK: blocking solution containing 1.5% bovine serum albumin (Merck) and antibodies diluted in 1.5% BSA + 0.1% Tween (Sigma Aldrich P9416). Secondary antibodies conjugated with fluorescent dyes (ThermoFisher Scientific) were diluted in 1% skim milk powder in CB and applied for 45 min (RT, darkness). Phalloidin labeling either with Atto488 or Atto633 (ThermoFisher Scientific) was applied together with secondary antibodies. Nuclei were counterstained with DRAQ5 (Cell Signaling 4084) and diluted in 1:1000 in CB or DAPI (NucBlue R37606, ThermoFisher) for 10 min (RT). Washing steps were performed with CB. Monolayered MCF10A cells were treated according to the same protocol with the following modifications. The fixation period was 10 min and cell membranes were permeabilized by 0.5% Triton-X 100-CB.

### 2.6. Confocal Microscopy

Live-cell imaging for TFM analysis was performed at 37 °C and 5% CO_2_ (cell incubator XL, Zeiss, Germany) with an inverse confocal laser scanning microscope (LSM880 with Airyscan detector) that used a 63× LD C-Apochromat water immersion objective (NA 1.15, Zeiss, Germany). Immunofluorescent-labeled fixed cells were analyzed with the same setup. A 40× LD C-Apochromat water immersion objective (NA 1.1, Zeiss) and a 63 Plan-Apochromat oil immersion objective (NA 1.4, Zeiss, Germany) were used. Images were taken and processed with Airy scan processing and maximum intensity projection (MIP) using the ZEN 2.3 black software (Zeiss, Germany). Live-cell imaging for acinar movement detection was carried out at 37 °C and 5% CO_2_ (cell incubator XL, Zeiss, Germany) using an inverted microscope (Axiovert 200, Zeiss, Germany), equipped with an AxioCam MRm camera (Zeiss, Germany) and an EC Plan-Neofluar 40× oil immersion objective (PH3, NA 1.30, Zeiss, Germany). Images were taken using the ZEN 2.3 blue edition software (Zeiss, Germany).

### 2.7. Actin Structure Analysis and Quantification

For CP analyses, acini were fixed, stained, and imaged with confocal microscopy, as described above. For lateral microspike quantification, several thin confocal image stacks (z-stacks) were taken, starting at 10 µm height above the cover slip and ending at the equatorial plane (defined as the cross-section with the largest diameter) of the acinus. The thickness of each z-stack was 1 µm, consisting of 5 images (optical section thickness: 247 nm). Individual z-stacks were merged as maximum intensity projections (MIP). In the MIP, the BM signal appeared as a red circle. The height interval between MIP images was 5 µm. For CP counting, MIP images were used. A single MS was defined as an actin structure that localized within the given BM scaffold (collagen type IV) and spanned at least the thickness of the collagen IV signal. The numbers of actin MS in each BM circle were divided by the respective BM perimeter (in µm). The BM perimeter was calculated from the measured BM radii. For actin structure analyses at the acinus–ECM interface, image stacks were taken and merged via MIP. The image stacks contained 7 to 15 images with an optical section thickness of 160 nm. MS and SF structures were detected. Therefore, the image was band-pass filtered; BP_img = G(I, 0.5) − G(I, 7) with BP_img as the resulting band-pass filtered image, I the maximum intensity projection, and G(I, X) the Gaussian filtered image of I with a filter kernel width of X pixel (pixel size 0.035 µm). The band-pass filtered image was then again smoothed by a Gaussian filter with a width of 1 pixel. Then, the histogram-based method of Otsu [28] was used to determine a threshold to create a binary mask for MS and SF. For each manually drawn cell border mask, the number and size (contact area) of the cells with MS and SF, as well as the MS coverage within cells, were calculated.

### 2.8. Traction Force Microscopy

Tangential substrate deformations caused by traction forces were visualized by tracking fluorescent beads. Beads (F8807 FluoSpheres, ThermoFischer Scientfic) were immobilized on the top of elastomeric substrates, as described elsewhere [29]. Maps of cell-induced traction stresses were calculated by regularized least-square fitting to the mechanical response of an elastic layer of 80 μm thickness on rigid substrates [30]. From these maps, strain energy (in femtojoule, fJ) was calculated as a scalar measure of overall mechanical activity. These calculations were based on previous work [30,31,32]. The required algorithms were implemented in MatLab (R2015a, The MathWorks Inc., and Natick, MA USA). Acini were analyzed for at least 18 h. All displacements (strains) and forces (stresses) were with reference to the first image of the series (*t* = 0). Therefore, we determined changes of strain energy with respect to that reference state. For spatio-temporal correlation of strain energy and actin SF pattern, a detection threshold was applied (≥25% of the maximum detected stress in nN/µm^2^) to segment hot spots. Manually drawn stress fiber masks were overlaid with hot spot segmentation for correlation.

### 2.9. Statistical Analyses

All measured values were plotted. For statistical data analyses mean values of each measured acinus were used instead, as indicated in the Figure legends. For data analysis of strain energy for each acinus per condition (*n* = 4), 60 values (in total 240 values of strain energy) were randomly selected using the bootstrap method (resampling) without replacement (1000 repetitions). For all analyses, the two-tailed nonparametric Mann–Whitney U-test with a 95% confidence interval was performed using GraphPad Prism version 8.4.2 (GraphPad Software, La Jolla, CA USA). *p*-values were defined as follows: n.s.: *p* ≥ 0.05; *: *p* < 0.05; **: *p* < 0.01; ***: *p* < 0.001; ****: *p* < 0.0001). 

## 3. Results

### 3.1. Tumor-like EGF and Substrate Stiffness Induce BM-Piercing Microspikes

To examine the formation of actin-based cell protrusions (CP) and their function for BM invasion, we used MCF10A-derived breast cell acini with an endogenously formed BM scaffold. This cell model has been widely used to study normal breast gland morphogenesis [33] and the invasion-promoting role of immature BM mechanics [6]. We adopted acini with either a low-developed BM (ld-BM, 11 days old) or a highly-developed BM (hd-BM, 22 days old) from the latter work. These cell models were used to investigate the interaction of mechanosensing CPs with differentially matured BM barriers. Of note, the mechanical strength of the BM barrier increases significantly during acinar maturation [4,5].

**Figure 1:**
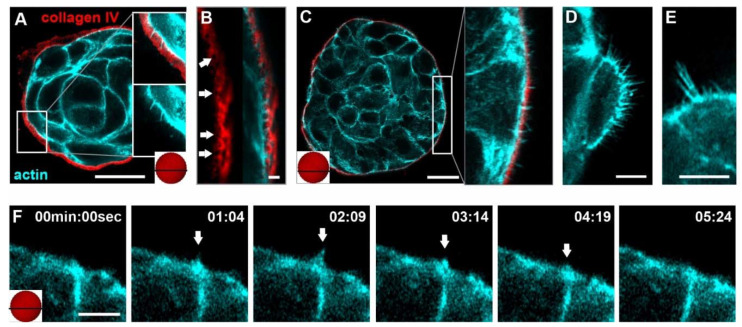
Breast acini form dynamic BM-breaching MS. The spatial localization of the analyzed confocal image is indicated as a black horizontal bar within the schematically shown red acinus (**A**,**C**,**F**). (**A**–**E**): MCF10A acini were fixed and IF-stained. Confocal images of breast acini treated with pro-invasive cues (24 h EGF treatment, glass). In all micrographs, collagen IV signal is shown in red and actin in cyan. (**A**) Overview of an EHS matrix-embedded hd-BM acinus with BM piercing CPs. (**B**) Magnification of an intact BM scaffold highlights the collagen IV network pores (white arrows) interleaved by short actin MS. (**C**) Heterogeneous arrangement and distribution of laterally elongated MS breaching the BM barrier. (**D**) A local hot spot of high MS density and (**E**) the formation of parallel MS bundles. (**F**) Image series of a living MCF10A/RFP-LifeAct acinus (EGF-treated). The image series depicts the lifetime of MS formation (white arrows) (min:sec). For complete image series, see Supplementary Movie S1. Scale bars: (**A**,**C**) = 20 µm; (**B**,**D**–**F**) = 5 µm.

Figure 1A shows that even the basal cell layer of hd-BM acini formed actin-rich, BM-piercing CPs at physiological ECM conditions. To recapitulate CP formation in pro-invasive conditions, we stimulated the acinar cells with soluble EGF. A sample transfer onto cover slips was mandatory to capture the dynamic process of CP formation and retraction with high microscopic resolution. Besides, such stiff substrate stimulation was purposely used in this work to trigger the invasive transition of benign breast acini, as previously described [6].

In experiments on ld-BM acini, we observed numerous actin-rich CPs. These acini showed pores in the collagen type IV network of the BM (Figure 1B), frequently penetrated by thin finger-shaped actin structures (Figure 1B). These condensed actin assemblies (lengths mainly < 2 µm) were precursors of more elongated CPs reaching deeper into the extracellular space. We defined these short CPs as microspikes (MS). Actin MS were heterogeneously distributed over the entire acinar surface (Figure 1C).

Moreover, local hot spots of high MS density (Figure 1D) and single MS with extended lengths of approx. five µm were evident (Figure 1E). The life-cycle of a single observed MS spanned several minutes (Figure 1F and Supplementary Movie S1). Two additional movies provide the complete visualization of this highly dynamic actin reorganization in several MS (Supplementary Movies S2 and S3).

**Figure 2:**
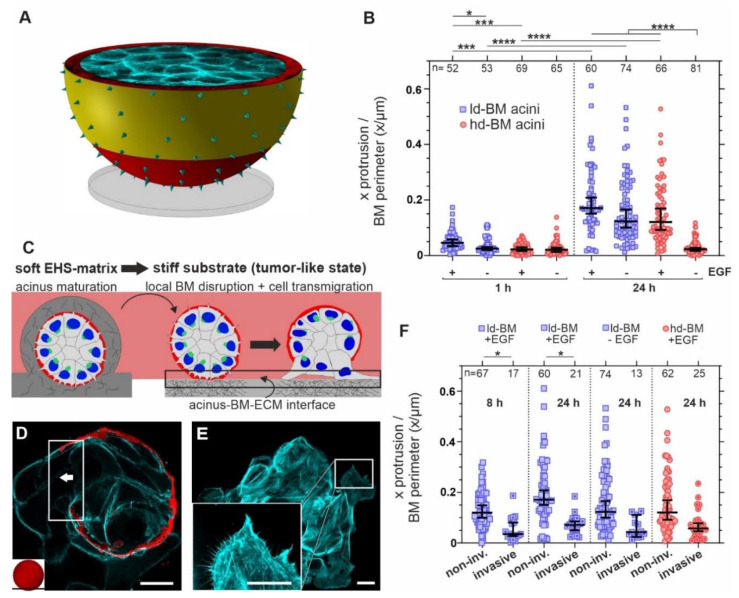
MS formation is fueled by tumor-like stiffness and oncogenic EGF. For analyses, MCF10A acini were fixed and IF-stained. (**A**) The schematic illustrates the acinar area analyzed for MS quantification (yellow area: start at 10 µm above the glass up to the equatorial plane, defined as the cross-section with the largest diameter). MS were counted for several confocal image planes in this area (see material and methods for a detailed description). (**B**) Scatter plot summarizes MS numbers in non-invasive acini depending on the BM state and oncogenic EGF treatment over time. Counts of protrusions (x) were divided by individual BM perimeters (in µm) of each analyzed confocal image. For a summary of observed acinar radii for BM perimeter calculation, see Supplementary Figure S1. (**C**) The experimental workflow of the designed invasion assay illustrates the invasive transition of benign acini transferred on a stiff substrate and conditional EGF stimulation (black box: area of invasion at the cell–BM–substrate interface) (**D**) A representative image shows local BM disruption (white box) and invasive cell transmigration (white arrow indicates the direction of cell migration) in an EGF-treated acinus. (**E**) Collectively outgrowing cell clusters showed frequent filopodial CP at the migration front. For additional images, see Supplementary Movie S4. (**F**) Scatter plot compares the lateral MS formation in invasive and non-invasive acini fractions depending on pro-invasive cues (24 h on glass). Sample size *n*: number of analyzed images of at least three independent experiments. Scatter plots: bars: median with 95% confidence interval. For statistical tests mean values per acinus were used: Mann–Whitney U-test with: **** = *p* < 0.0001, *** = *p* < 0.001, * = *p* < 0.05. Scale bars: (**D**,**E**) = 20 µm.

Next, we aimed to quantify the MS formation frequency depending on BM maturation, EGF stimulation, and adhesion time on a stiff substrate. The MS signals were detected over a defined area of the acinar surface. The MS number per BM perimeter was calculated (Figure 2A—yellow area). This approach revealed a significantly increased MS formation due to temporally extended EGF stimulation (Figure 2B). In detail, after one hour, no differences were detectable between EGF-treated and untreated hd-BM samples. Only ld-BM acini were affected by such brief EGF stimulation. The fold change (FC) of MS formation was 1.8. The EGF effect was pronounced upon prolonged stimulation. After 24 h, EGF significantly induced MS formation in ld- and hd-BM acini, whereas the untreated counterparts remained at the basal level that was measured after one hour (FC = 5.3).

Beyond the strong effect of EGF on ld-acini (0.17 protrusion/µm corresponding to a median of 45 MS per BM perimeter at 24 h), substrate stiffness played a role; even in the absence of EGF stimulation, since immature ld-BM acini exhibited raising CP numbers with increasing adhesion time (FC = 4.9) (Figure 2B). Together, these results indicate that soluble EGF fueled MS formation in pre-invasive breast acini. In addition, acini with mechanically matured and thicker BM appeared unaffected by stiff adhesion substrates compared with EGF-lacking ld-BM acini (Figure 2B).

We next investigated the protrusion formation at the late stage of invasive transition in acini that already underwent local BM disruption and cell transmigration. Figure 2C illustrates the general invasion process after seeding non-invasive breast acini on stiff substrates and the start of EGF stimulation. After this switch of ECM conditions, the BM got eventually disrupted at the acinus-ECM contact area (Figure 2C, black box) and cells collectively transmigrated from the acinar body into the microenvironment (Figure 2D). The leading edges of these outgrowing cells contained a high density of elongated filopodia-like protrusions—a characteristic of cells migrating on planar substrates (Figure 2E and Supplementary Movie S4). Surprisingly, the basal cell layer of acini that underwent local BM disruption formed fewer lateral CPs than cells of the non-invasive fractions (Figure 2F). This unexpectedly low number was irrespective of EGF treatment and BM state (mean FC = 2.7) (Figure 2F).

### 3.2. Invasive Acini Switch from Non-Proteolytic to Proteolytic Invadopodia

**Figure 3:**
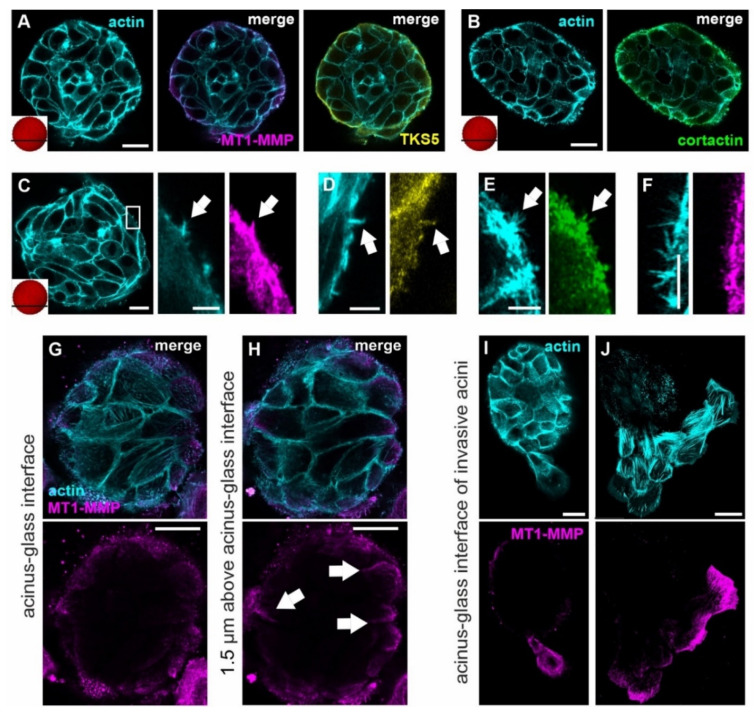
Subcellular localization of invadopodia markers in acinar and invasive cells. MCF10A acini were fixed and IF-stained. (**A**–**F**): Confocal images of pre-invasive breast acini with intact BM treated with pro-invasive cues (ld-BM, 24 h EGF treatment, glass). (**A**,**B**): The actin cortex (cyan) of the basal cell layer of pre-invasive acini co-localizes with the invadopodia marker proteins MT1-MMP (magenta), Tks5 (yellow), and cortactin (green) (for single MT1-MMP, TKS5, and cortactin signal see Supplementary Figure S2A. (**C**–**E**): Visualization of the actin cytoskeleton with small single actin-based lateral MS, co-localized with MT1-MMP (**C**), Tks5 (**D**) and cortactin (**E**). (**F**) Area of high MS density without co-localization of MT1-MMP and actin MS. (**G**,**H**) MT1-MMP protein localization at cortical actin structures in cells at the BM–substrate interface (cf. **A**) (white arrows). (**I**,**J**): Micrographs showing MT1-MMP at the migration front of invasive cells after BM breakdown. Antibody control staining is provided in Supplementary Figure S2D. Scale bars: (**A**–**C**,**G**–**J**) = 20 µm, magnifications in (**C**–**F**) = 5 µm.

Pro-invasive ECM conditions induced MS formation in the basal cell layer, especially in low-matured acini. We next tested whether these protrusions contributed to invasion by proteolytic BM-weakening under most invasive conditions, i.e., ld-BM acini, EGF stimulation, and stiff substrate. The co-localization of three representative invadopodia marker proteins, namely Tks5, cortactin, and the membrane-bound MT1-MMP was analyzed. It turned out that all three markers accumulated within the actin cortex of invasion-triggered breast acini with intact BMs (Figure 3A,B and Figure S2A). Despite the signal in the basal actin cortex, only a few MS showed an actin co-localization with these invadopodia markers (Figure 3C–E, white arrows). The majority of CPs formed by acinar cells lacked, for instance, the proteolytic enzyme MT1-MMP. To this end, a representative image shows that co-localization of MT1-MMP with actin MS was occasional (Figure 3F).

A closer look at the basal cell layer, approximate to the underlying substrate, revealed MT1-MMP protein accumulation at the actin cortex (Figure 3G,H, white arrows) similar to that found at the outer rim of the entire basal cell layer (cf. Figure 3A). Striking evidence for the involvement of MT1-MMP was observed at the later stages of BM invasion. Here, MT1-MMP accumulated strongly in the leading cell that firstly transmigrated through the disrupted BM (Figure 3I). Further increased localization of MT1-MMP at the leading edges of the collectively invading cell cluster was detected (Figure 3J). ECM-spreading cells also formed abundant and elongated CP that stained positive for all IP markers (Supplementary Figure S2B,C).

Together, these results demonstrated that non-invasive breast acini feature a basal level of MT1-MMP. However, in pre-invasive stages, BM-breaching MS mostly did not contain the BM-degrading MT1-MMP. In contrast, at late stages of invasion, invadopodia formation with abundant MMP expression occurred in spreading cells that transmigrated into the ECM. Interestingly, we found fibrous and dot- or spike-shaped actin structures in cells that directly contacted the BM–ECM interface (Figure 3G). These structures clearly differed from the cortical actin cytoskeleton of basal cells that were localized elsewhere within the acinar body (Figure 3A).

### 3.3. Breast Acini Switch Cytoskeletal Organization at Sites of Potential BM-Invasion

**Figure 4:**
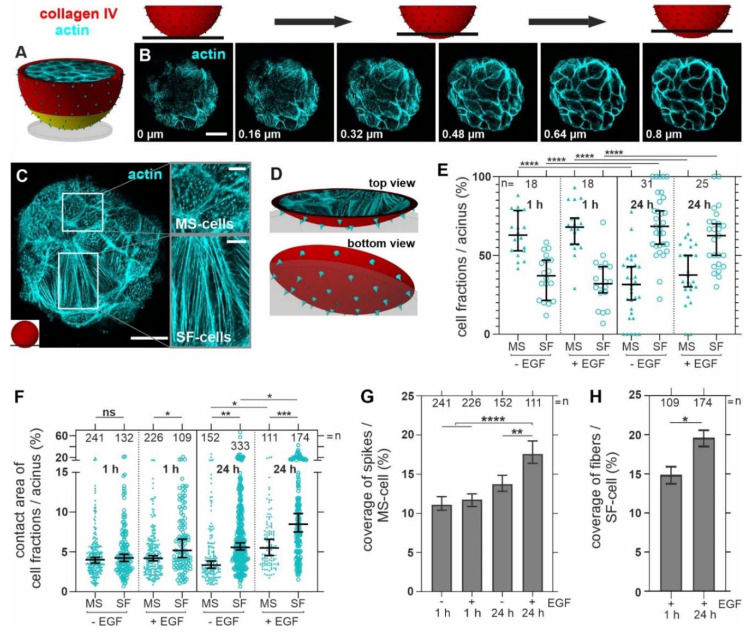
Tumor-like substrate stiffness and EGF trigger actin remodeling at the acinus-BM–ECM interface. Ld-BM acini were treated with tumor-like conditions (+/− EGF for 1 and 24 h on stiff substrate) and subsequently fixed and IF-stained for analyses. (**A**) The schematic illustrates the analysis area at the cell–ECM interface (yellow). (**B**) Confocal image series shows the basal acinar cell layer (1 h on glass, −EGF). (**C**) A representative image shows the actin networks in MS and SF cells (1 h on glass, +EGF). (**D**) The schematic illustrates the observed spatial orientation of actin dots, spikes and fibers. (**E**) A scatter plot for MS and SF cell formation at cell-ECM contact area (basal cell layer of an acinus as shown in (**C**)) depending on substrate stiffness and duration of EGF stimulation. Cell fractions (%) were calculated by dividing the MS and SF cell numbers by the total cell number at the contact plane of individual acini. (**F**) A scatter plot for the size of MS and SF cells at the contact area depending on the duration of contact to a stiff substrate and EGF stimulation (*n* = all analyzed single cells/cell fractions of the observed acini in (**E**)). The contact area (%) of cell fractions was calculated by dividing the cell area (pixel) of MS and SF cells by the total contact area of individual acini (pixel). (**G**,**H**) The graphs show microspike coverage in MS cells (**G**) and stress fiber coverage in SF cells (**H**) depending on tumor-like ECM. *n* = total number of analyzed acini (**E**) or cells (**F**–**H**). Scatter plots and bar charts (**E**–**H**): bars: median with 95% confidence interval. For statistical tests mean values per acinus were used: Mann–Whitney U-test with: n.s.: *p* ≥ 0.05; *: *p* < 0.05; **: *p* < 0.01; ***: *p* < 0.001; ****: *p* < 0.0001. Scale bars: (**B**,**C**) = 20 µm.

To investigate cytoskeletal remodeling in invasion-triggered breast acini, we carefully analyzed the cell–BM–ECM contact zone of pre-invasive acini before local BM-disruption occurred (Figure 4A, yellow area). Figure 4B shows the characteristics of actin cytoskeleton networks formed by the first BM-covered basal cell layer at the contact plane to the underlying substrate. Based on the predominant actin structures, we defined two cell phenotypes (Figure 4C). Firstly, microspike (MS) cells exhibited a high density of actin dots and spikes that often appeared towards the underlying substrate plane. Secondly, stress fiber (SF) cells were covered by ventral actin fibers orientated parallel to the substrate plane (illustrated in Figure 4D).

Interestingly, the inter-acinar proportion of MS and SF cells significantly changed with prolonged incubation on stiff substrates and EGF stimulation. In detail, directly after seeding (1 h), MS cells dominated the basal cell layer (Figure 4E). However, after 24 h, the opposite held true, as the SF cell fraction was significantly increased and dominated over MS cells. This switch from MS to SF was related to the stiff substrate and further unaffected by additional EGF treatment (Figure 4E).

Further, the total number of substrate contacting cells per acinus was reduced over time by 18% (mean cell number of cells in contact at 1 h = 20 and 24 h = 14 cells of in total 36 (1 h) and 56 (24 h) analyzed acini). At the same time, the contact area of individual SF cells increased significantly compared to MS cells (Figure 4F). The rapid spreading of SF cells after one hour was dependent on EGF treatment (Figure 4F). Besides the overall decrease in MS cells after 24 h, a significant increase in microspike coverage in the remaining fraction was evident. EGF also gradually enhanced the stress fiber coverage in the SF cell fraction (Figure 4G,H).

Together, these results indicate a switch from MS to SF cells in tumorous ECM conditions. Furthermore, this fundamental cytoskeleton reorganization at the basal cell cortex, i.e., an increase in the number of SF cells and the contact area, was reinforced by pro-invasive EGF and preceded the temporally increased formation of actin microspikes.

### 3.4. Actin Microspikes and Stress Fibers Interact with the BM Scaffold

**Figure 5:**
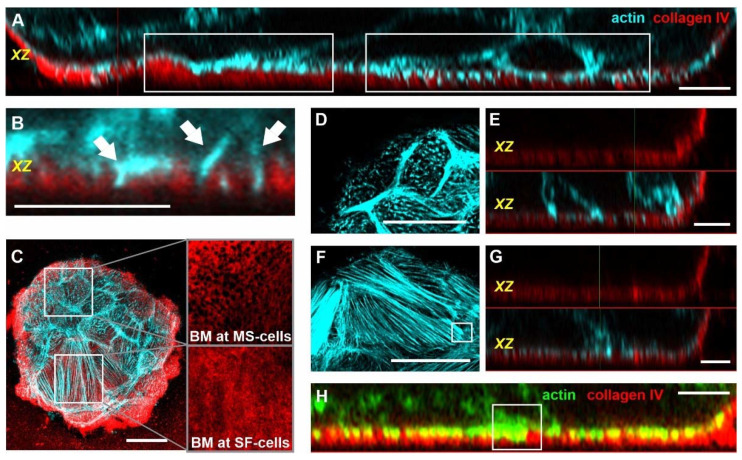
The actin cytoskeleton intercalates with the BM scaffold. Pre-invasive MCF10A acini were fixed and IF-stained. (**A**) The orthogonal projection of the basal cell layer that is in direct contact with the underlying substrate (not visible) highlights the connections between actin (cyan) and the BM (collagen IV, red). Left box: F-actin located on top of collagen IV network. Right box: F-actin penetrating the collagen IV network. (**B**) The micrograph shows BM traversing microspikes (white arrows) in an MS cell of an EGF triggered ld-BM acinus (7 h on glass). (**C**) Overview image of the acinus–BM–substrate interface shows different BM porosity beneath MS cells (upper box) and SF cells (lower box) (ld-BM, 1 h on glass, –EGF) (same sample analyzed as in Figure 4C) (**D**) Acinus with MS cells (ld-BM, 1 h on glass, –EGF). (**E**) The orthogonal projection of MS cells shows actin-rich microspikes penetrating the BM collagen IV scaffold. (**F**) Acinus with SF cells (ld-BM, 24 h on glass, +EGF). (**G**) The orthogonal projection of SF cells shows actin stress fibers ends at the top of the collagen IV network without detectable penetration. (**H**) The orthogonal projection of an SF cell highlights a BM-penetrating stress fiber-tip (cf. box in (**F**)). Scale bars: (**C**,**D**,**F**) = 20 µm, others = 5 µm.

The basal cell layer consists of characteristic MS and SF cells. We next asked how these structurally distinct cell populations interact with the BM barrier that separates them from the surrounding extracellular space. The analysis of pre-invasive acini with intact BM revealed two modes of cell–BM interaction. Firstly, contact areas were evident where ventral filamentous actin structures appeared as a horizontal layer on top of the collagen IV network (Figure 5A, left box). Secondly, areas with actin spikes intercalating with the BM scaffold were frequently detectable (Figure 5A, right box). Here, elongated actin microspikes that reached the underlying glass substrate were frequent (Figure 5B, arrowheads). A closer investigation of the BM’s collagen IV meshwork showed that basal cells with high MS density located onto porous BM areas (Figure 5C, upper box). In contrast, SF-rich cells adhered to a less porous regions of the collagen IV meshwork (Figure 5C, lower box).

To further evaluate the dynamics of actin–BM interactions, we used pro-invasive EGF to trigger the cytoskeletal switch. In line with our previous results (cf. Figure 4E), after a short one-hour treatment, mostly MS cells with a high actin spike coverage were present (Figure 5D,E). Interestingly these MS cells showed frequent occurrences of BM pore penetration (Figure 5E). On the other hand, after 24 h of EGF treatment, the frequency of BM traversing actin spikes was reduced in SF cells (Figure 5F,G). As described above, most of the ventral actin structures in SF cells arranged on top of the BM (Figure 5H).

In SF cells, BM penetration occurred at the ends of ventral stress fibers ( cf. white boxes in Figure 5F,H). Together, our results imply that cells in breast acini penetrate the BM by frequently formed actin spikes and by ventral stress fiber tips. The frequencies of these protrusive modes switch during the cytoskeletal remodeling process at the cell–BM–ECM interface.

### 3.5. Acinar Cells Adhere to the BM and the ECM by Force-Transmitting FAs

**Figure 6:**
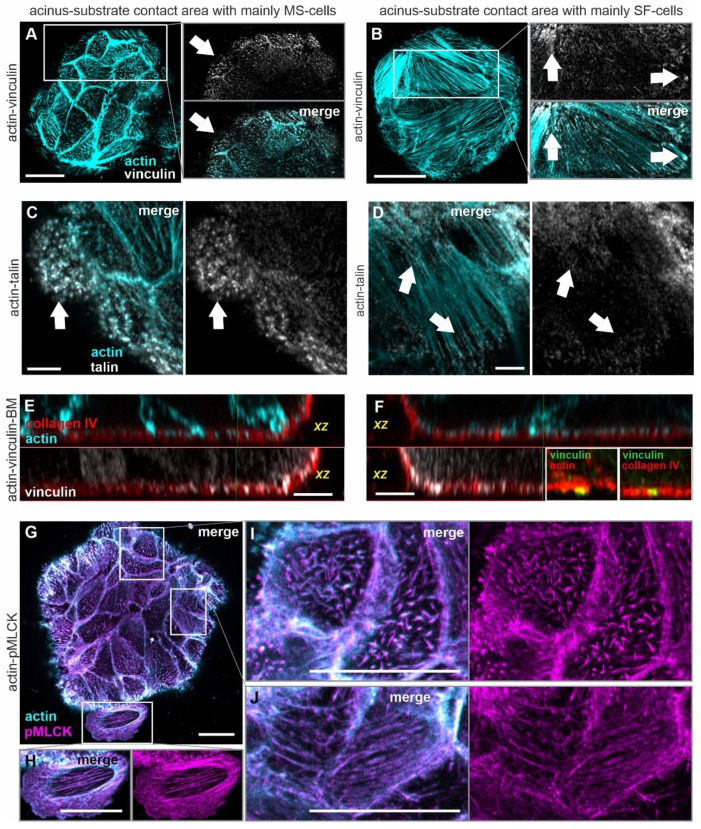
Focal adhesion-mediated actomyosin activation in MS and SF cells. MCF10A acini were fixed and IF-stained. (**A**–**D**): Representative confocal images of the acinus–BM–substrate interface. (**A**,**C**) MS cells (1h on glass, −EGF) and (**B**,**D**) SF cells (24 h on glass, +EGF) show the co-localization of actin microspikes and stress fiber ends with vinculin and talin (arrowheads) ((**A**,**B**): same sample analyzed as in Figure 5D,F). (**E**) The orthogonal cross sections highlight the co-localization of vinculin with microspikes in MS cells. (**F**) SF cell with co-localization of vinculin with stress fiber ends within the BM scaffold (yellow). White box: position as indicated in ((**B**), right arrow) (collagen IV and actin signal confocal images in (**E**,**F**) are reused from Figure 5E,G). (**G**) Overview image of the acinus–BM–substrate interface demonstrates pMLCK-actin co-localization. (**H**) Zoom-in on an invasive cell shows actin stress fiber and pMLCK co-localization after BM transmigration. (**I**) Detailed view on pMLCK bound to actin microspikes in MS cells and (**J**) to stress fibers in SF cells. A pMLCK staining of cells on a planar substrate and a secondary antibody control staining is provided in Supplementary Figure S3B,C. Scale bars in (**A**,**B**,**G**–**J**) = 20 µm and (**C**–**F**) = 5 µm.

MS and SF cells form actin microspikes and stress fibers that co-localize with the BM scaffold. We tested how acinar cells were coupled to the underlying BM and ECM. We found that MS and SF cells formed FAs. The mechanosensory FA proteins vinculin and talin were frequently associated with microspikes in the cell cortex of MS cells (Figure 6A,C, white arrows) and with stress fiber tips in SF cells (Figure 6B,D, white arrows). However, the FA-patches at fiber tips were generally smaller in non-invasive acinar cells than in spread cells that underwent BM invasion (Supplementary Figure S3A). Vinculin spots were present within the collagen IV network in both cell fractions. However, in MS cells, vinculin was exclusively found in BM-piercing actin spikes (Figure 6E). In SF cells, vinculin predominately co-localized with stress fiber tips anchored within the collagen IV scaffold (Figure 6F, cf. right arrow in B).

When seeded on planar substrates, breast acini perform a continuous coherent movement (see Supplementary Movie S5) that is essential for acinar differentiation and homeostasis [34]. We next addressed how FA-bound MS and SF structures are involved in actomyosin contractility and acinar cell motion triggered by pro-invasive conditions. To this end, we analyzed the localization of activated pMLCK with cytoskeletal actin as an indicator for force-transmitting actin fibers. Figure 6G demonstrates a clear pMLCK-actin co-localization in the entire basal cell layer at the BM–substrate interface. In detail, activated pMLCK was bound to actin microspikes in MS cells and actin stress fibers in SF cells (Figure 6I,J). Furthermore, the overall pMLCK intensity in pre-invasive acinar cells was comparable to the one in spreading and invasive cells (Figure 6H). Together, these results indicate that stress fibers and microspikes were mechanically coupled to the underlying BM and the substrate via mechanosensory FAs. It appears likely that these FAs transmit actomyosin-driven mechanical stress between the BM scaffold and the ECM.

### 3.6. Tumor ECM-Induced SF Cell Formation Drives Myosin II-Mediated BM Stress

Finally, we tested how the switch from MS to SF cells contributes to physical BM disruption. Placing non-invasive MCF10A breast acini onto tumor-like elastomeric substrates (12 kPa) induces the invasive transition characterized by local BM disruption and cell dissemination [6]. Quantitative cell traction force microscopy was applied to measure cell force-based BM stress at the acinus–BM–substrate interface.

**Figure 7:**
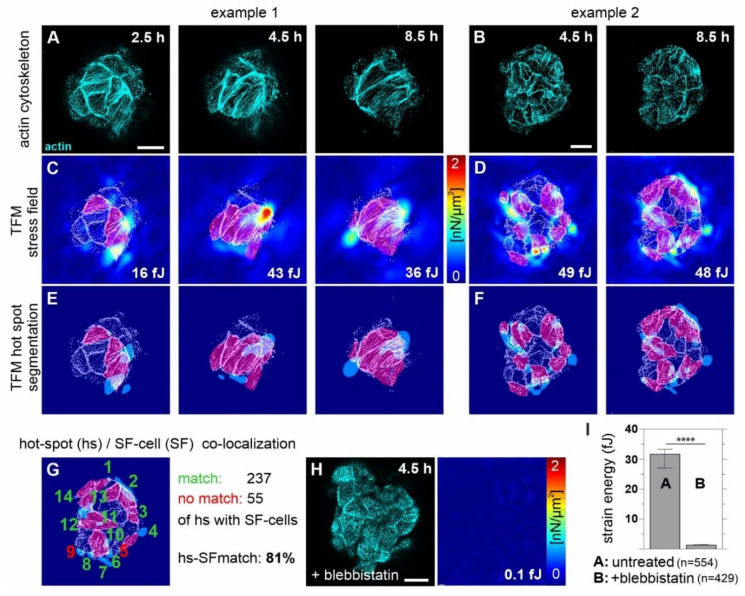
SF cells of invasion-triggered breast acini exert force hot spots on the acinus–BM–ECM interface. TFM image sequences show mechanical stress at the BM–substrate interface exerted by MCF10A/RFP-LifeAct acini (ld-BM, +EGF) on 12 kPa substrates over time. (**A**,**B**) Confocal images of the acinus–ECM interface representing the actin cytoskeleton. (**C**,**D**) Matching stress maps (linear lookup table for pseudo-color display) used to calculate total strain energy within the field of view (indicated by white numbers in (**C**,**D**,**H**)). SF cells were marked manually (magenta). The stress map at one hour for Example 1 is provided in Supplementary Figure S4A. (**E**,**F**) Hot spots (hs) in stress maps (light blue patches) were segmented automatically and counted for the spatial correlation with SF cells. (**G**) The representative image illustrates the counting procedure and the quantitative result of hot spots and stress coherence (in total 292 hot spots of four independent analyzed acini (30 images)). Hs segmentation (light blue patches) and counting (green and red numbers) was carried out every two hours for each acinus within a measuring period of 18–20 h. The localization of 237 hot spots matched with SF cells (green numbers), and 55 did not (red numbers). For a further example of matching hot spots with SF, see Supplementary Figure S4C. (**H**) Representative acinus with MS cell treated with blebbistatin (25 µM). Left: actin cytoskeleton, right: TFM stress map (for additional time points, see Supplementary Figure S4B). (**I**) Calculated strain energies (SE) of acini treated with blebbistatin compared to untreated samples. The graph shows the SE values measured over a period of 18–20 h (images were taken every seven minutes). Bars show median with 95% confidence interval; Untreated acini: median SE = 32 fJ (*n* = 4, in total 554 images); Blebbistatin treated acini: median SE = 1.4 fJ (*n* = 4, in total 429 images); DMSO control (not shown): median SE = 45 fJ (*n* = 3, in total 438 images). Mann–Whitney U-test with: **** = *p* < 0.0001. Scale bars: 20 µm. For complete image series of the actin dynamics, see Supplementary Movies S6 and S7.

Image sequences showed the dynamic change in actin cytoskeleton organization in moving MS and SF cells at the substrate contact plane (Figure 7A,B). Forces exerted by these cells caused spatially confined hot spots of substrate stress fields (Figure 7C,D). These stress hot spots appeared at places where actin stress fiber tips were present. Two representative samples highlight two principal SF cell phenotypes with different stress fiber organizations generally found in breast acini:

Example 1 illustrates the formation of large elongated SF cells. Here, the same stress fiber orientation propagated between neighbored cells. The corresponding significant stress hot spots localized at the borders of these cells (Figure 7A) indicated high cell contractility along the axes of stress fibers. In contrast, no stress hotspots were detectable underneath MS cell populations at an early time point (one hour), before SF cell formation started (Figure 7A,C).

Example 2 exhibited a small SF cell phenotype with more dispersed, thinner, and more abundant actin stress fibers. These fibers had less-ordered orientations than in sample 1. Accordingly, stress hot spots were less pronounced (Figure 7B,D). However, the enhanced number of these spots resulted in comparable total strain energy (SE) of 43–48 fJ for both SF cell phenotypes at different measuring points (4.5 and 8.5 h). Strain energy is a robust measure of cell mechanical activity, calculated from substrate deformation and stress maps.

Stress hot spots were localized automatically by segmentation to facilitate comparison with actin localization (Figure 7E,F). This result demonstrated the co-localization of high-force amplitudes and SF cells. In addition, the quantification revealed a significant match (81%) between stress hot spots and SF cell localization (Figure 7G). Whereas not every stress fiber transmitted detectable forces, overall hot spot formation was associated significantly with SF cell formation.

To finally prove the functional link between SF cells and contractility-driven BM stress, we pharmacologically inhibited myosin II activity in invasion-triggered breast acini. Untreated acini exerted high-force amplitudes after 4.5 h, while myosin inhibition led to a complete loss of substrate deformation, with residual strain energy levels that were within the limits of background noise. Most importantly, the absence of force accompanied the lack of SF cells (Figure 7H,I and Figure S4B).

Together, our results functionally link the EGF- and stiffness-induced switch from MS to SF cells with cell contractility. At the acinus–BM–ECM interface, these forces cause mechanical BM stress that finally contributes to the invasive transition of benign breast cells.

## 4. Discussion

This study demonstrates that non-malignant breast acini form highly dynamic actin-based protrusions. The importance of such filopodial protrusions for single-cell migration and ECM invasion is well known [12,35]. Here, we investigated acini at physiological conditions and consistently observed actin-rich protrusions that traversed the BM. In view of the demonstrated functions of actin-rich protrusions in single-cell interactions with the ECM, we see a high probability that these BM-traversing protrusions also serve as ECM-sensing elements. Furthermore, the life cycle and kinetics of acinar CPs matched those of mechanosensory actin protrusions previously found in single MCF10A cells testing planar substrate rigidity [36].

Our experiment design was tailored for the investigation of the cell–BM–ECM interface at high spatial resolution. Thus, we omitted matrix embedding that would have hindered light microscopic detection of the tiny actin structures. The lack of matrix adhesion motifs could explain the absence of elongated lateral protrusions of the basal cell layer at a distance from the cell–BM–substrate interface. Concordantly, previous work showed that breast acini form elongated ECM-penetrating protrusions when embedded in a hydrogel matrix [6]. Further, the fast retraction and degradation cycle of short lateral CPs underpinned the need for ECM adhesion sites for protrusion stabilization [37]. Overall, it appears that acinar CPs bridge the BM scaffold to probe the ECM. The use of stiff glass substrates was a prerequisite for high-resolution imaging of the cell–BM–substrate interface. For TFM, we used rigid elastomeric substrates (12 kPa). Of note, the stiffness of both materials differs over several orders of magnitude. However, previous work demonstrated that BM disruption and invasiveness of MCF10A breast acini were strongly induced by 12 kPa and glass substrates [6].

It has been demonstrated that both oncogenic EGFR signaling and tumor ECM resembling substrate stiffness initiate the invasive transition of benign and originally non-invasive breast gland acini [6,38]. We used this paradigm to study the functional role of BM-piercing actin protrusions for the pathological process of BM disruption and cell transmigration. Our data imply an increased formation of actin protrusions upon tumorous ECM cues, i.e., soluble EGF and substrate stiffening. In that respect, low-matured breast acini with mechanically weak BM scaffold were most responsive and exhibited the highest induction of actin protrusions. This result parallels the previously described high invasiveness of ld-BM acini compared to highly-maturated hd-BM acini [6]. Thus, we used these highly protrusive ld-BM acini to study the pro-invasive actin remodeling mechanisms at the cell–BM–ECM interface.

Breast cancer-related EGF stimulation is strongly associated with poor prognoses and mediates invadopodia precursor formation in breast cancer cells [39,40,41]. Further, an increased actin protrusion density correlates with increased malignancy of MCF10ADCIS and MCF10AT cell variants [42]. The observed enhanced protrusion activity could hence indicate an amplified trans-BM ECM sensing during tumor cell invasion. Together, our data suggest a functional coupling between the increased formation of BM-piercing actin protrusions and high invasiveness of breast acini. Besides, we found an overall reduction in actin CPs within invasive breast acini. This intriguing finding points at a cell-to-cell communication network that wires the entire acinar basal cell layer. Clearly, further work is needed to unveil such communication mechanisms within epithelial breast cell clusters.

The BM scaffold of the breast epithelium is constantly remodeled. This process is essential for the differentiation and maintenance of tissue integrity [34,43]. Accordingly, we found a basal MT1-MMP level at the plasma membrane of the basal acinar cell layer. These cells contact the BM and, most probably, contribute to the MMP-driven BM remodeling process. Interestingly, BM-piercing protrusions in the same cells mostly lacked the proteolytic MT1-MMP, besides rare exceptional events. Importantly, MT1-MMP activates proMMP-2 and -13 that mediate collagen degradation [44]. The lack of MT1-MMP argues for the absence of such active MMP cascades in actin protrusions.

Furthermore, the formation of elongated BM-piercing actin protrusions in breast acini does not depend on cancer-related secreted MMP-1, -3, -7, -9 and cell membrane-bound (MT1-MMP) [6]. The BM’s structure-forming collagen type IV meshwork exhibits pore sizes mainly in the range from 9–100 nm [4]. Together, these facts indicate that actin microspikes with small diameters in the 100 nm range can intercalate into already existing pores of the BM, even in the absence of enzymatic degradation. After initial penetration by a thin microspike, subsequent mechanical widening of the original BM pores by actin reinforcement and thickening of the initial protrusion could ease further growth. So far, pore widening has been reported only for fibrillar collagen type I matrix invasion of breast cancer cells via MT1-MMP lacking invadopodia [45]. We propose that benign breast acini use similarly expanding actin protrusions to efficiently traverse the sheet-like collagen type IV meshwork of a dense BM barrier for ECM probing.

Invasive cells are characterized by actin-rich invadopodia, although various shapes and molecular compositions finally define their functionality [13]. Nevertheless, invadopodia are widely known for MMP-dependent matrix degradation [46]. In contrast to this classical paradigm, we show that tumor ECM-triggered breast acini mostly exhibit actin protrusions that mostly lack any detectable MT1-MMP protein localization. Furthermore, the low detection intensity of the invadopodia markers MT1-MMP, Tks5 and cortactin [47,48], and Supplementary Figure S2 indicates non-proteolytic invadopodia precursors. Such protrusions are formed at the early stages of invasive transition when the acinus and the BM are still intact. However, as witnessed by the presence of spurious amounts of MT1-MMP (cf. Figure 3G,H), the small fraction of invadopodia-like MS at the basal cell layer most probably relates to a fine-tuned proteolytic BM remodeling.

Particularly at the cell–BM–ECM interface, i.e., the hot spot of potential BM degradation, MT1-MMP was exclusively located at the plasma membrane, as described for the entire basal acinar cell layer (cf. Figure 3A,H). Our finding contrasts to previous work that characterized such dot-like actin structures as MT1-MMP positive invadopodia of single invasive breast cancer cells [49]. However, we can rule out technical limitations because other punctual protein patches (vinculin and paxillin) were detectable at the cell–BM–ECM interface, and MT1-MMP was highly visible in cells that had left the acinus already. This contradictory result highlights that single migrating tumor cells and epithelial cells of non-malignant BM-covered breast acini use different contextual invasion programs. In line with this, we observed massively increased numbers of MT1-MMP positive invadopodia in acinar cells that transmigrated into the microenvironment. Reduction in cell–cell contact, polarization loss, cytoskeleton reorganization, and invadopodia stabilization are hallmarks of epithelial-to-mesenchymal transition (EMT) [50]. Our results imply that EGF-triggered acinar cells could gain such EMT plasticity to efficiently transmigrate from the acinar body and spread into the microenvironment.

The existence of MMP-independent, force-driven BM breaching programs that fuel mouse embryogenesis and breast cancer invasion are well documented [6,43,51]. However, it remained poorly understood how actin cytoskeleton reorganization drives non-proteolytic BM disruption. The present work, therefore, focuses on cell–ECM interactions across a continuous, still-intact BM barrier. In line with our finding of BM traversing actin protrusions, ATP-enriched filopodia have been associated with ATP-fueled mechanical BM cell transmigration in *C. elegans* [1]. We furthermore discovered a fundamental cytoskeletal adaption in human breast acini. While cortical actin networks characterized the acinar cell body, actin stress fibers were prominent at the stiff acinus–BM–ECM interface. Actin structures steer mechanotransduction, cellular tension, and cell function [52]. The spatially confined actin network remodeling at the BM–ECM interface could hence mediate invasive behavior.

Epithelial cells are bound to the BM primarily by laminin-binding hemidesmosomes (HD) that connect to the intermediate filament network [53]. The motile phenotype of invasive cells requires the disassembly of stable type I HD and the assembly of dynamic type II HDs or the formation of BM-binding focal adhesions [54]. Here, we describe for the first time that human breast acini cells are mechanically anchored to the BM by force-transmitting FA complexes. At these sites, MS structures and the ends of ventral SFs interlink with mechanosensory FA proteins. This result suggests that both MS and SF contribute to (trans)-BM sensation.

The collagen network of the BM forms a continuum with the surrounding ECM. In our experimental setup, the BM physicochemically couples with the BM protein functionalized substrate. Thus, we propose that acinar cells can sense substrate stiffness indirectly via BM-bound FA complexes, as well as via BM-piercing filopodia. However, the molecular BM-binding partners of these specific BM adhesions remain unknown.

A significant discovery of this work was the progressive appearance of actin stress fiber-rich cells at the ECM contact plane that accompanied the decrease in cortical actin bundles. Such a mechanoresponse of breast cells to pro-invasive ECM conditions is supported by recent work that demonstrated the loss of cortical actin and stress fiber formation during EGF-induced EMT of non-tumorigenic rat liver cells [9]. Together, we propose that MS to SF remodeling by EGF and ECM stiffness induces EMT-like cell plasticity that non-tumorigenic human breast acini undergo as a very early event in invasive transition.

We investigated the relationship between actin SF formation and pro-invasive cell force generation. We measured cellular traction forces directly at the BM–ECM contact plane to functionally link actin remodeling with pro-invasive cell contractility. Elastomeric substrates with an elastic modulus of 12 kPa resembled breast tumor rigidity [55]. Strain energy was calculated from the substrate deformation fields as a robust measure for the force acini exerted through the BM barrier on the substrate, as described previously [6].

An important key finding was that cells packed with ventral stress fibers at the acinus–BM–ECM interface matched spatiotemporally with hot spots of high cellular traction. Ventral stress fibers are typically anchored to FAs at both ends and are essential for matrix adhesion and cell contractility [56]. Concordantly, previous work has demonstrated that EGF promotes stress fiber and FA formation via tensin-DLC1-RhoA signaling, resulting in highly migratory MCF10A cells [57]. Our work demonstrates for the first time that EGF and ECM stiffening induces the formation of actin stress fiber-packed cells in a BM-covered breast acini model.

More than two decades ago, ground-breaking work showed the functional relation of MLCK phosphorylation, SF and FA formation, and contractility in fibroblasts [58]. Our work now demonstrates the strong co-localization of activated phosphorylated myosin light chain kinase (pMLCK) with ventral actin stress fibers in breast acini. Moreover, inhibition of myosin-ATPase activity led to a complete lack of actomyosin-driven contractility at the BM–ECM interface prone to cell invasion. These new results indicate that the high contractility of BM-covered and invasion-triggered breast acini strongly depends on myosin II activity. Interestingly, MLCK has catalytic and actin-scaffolding functions, with opposing effects on cell behavior. While breast cancer-related loss of MLCK expression increases invasiveness [59], the inhibition of its catalytic domain leads to decreased cell migration [60]. The latter function supports our finding that pMLCK catalyzes actomyosin contractility during the invasive transition of benign breast cells.

Since pMLCK co-localized with actin microspikes, we hypothesize that these structures exert vertical forces towards the underlying substrate. Of note, the applied TFM assay measures tangential forces but is insensitive for vertical substrate deformations. However, ECM degrading and pushing invadopodia have been found in invasive head and neck carcinoma cells [19]. Moreover, high amplitudes of protrusion pushing and pulling forces correlate with mechanical BM disruption in MCF10A breast acini [6]. Together, these findings suggest that BM-anchored and mechanosensitive actin microspikes could contribute to force-driven BM stress at a very early invasive stage. Future work should address the underlying cellular regulation circuits that modulate myosin activation in actin microspikes during early EMT transition. In later stages, the increasing frequency of highly contractile stress fiber-rich cells progressively increases BM stress amplitudes.

In physiological environments, acinar cell motion essentially regulates BM maintenance and homeostasis [34]. In contrast, the same movement of EGF-triggered SF cells at the BM–ECM interface with tumor-like stiffness leads to imbalanced solid stresses. The BM barrier finally fails to compensate for these chronic stresses, BM disruption, and cell transmigration results [6]. Proteolytic invadopodia contribute to this BM-weakening and disruption process [2,21,61]. Importantly, our data showed that breast acini activate predominately non-proteolytic but mechanically active protrusions at the early stages of BM invasion. MT1-MMP-driven mechanisms are activated later in cells that disseminate into the ECM. This functional switch of protrusions is mediated by EMT-like actin cytoskeleton reorganization, finally inducing myosin II-derived mechanical BM stress.

## 5. Conclusions

**Figure 8:**
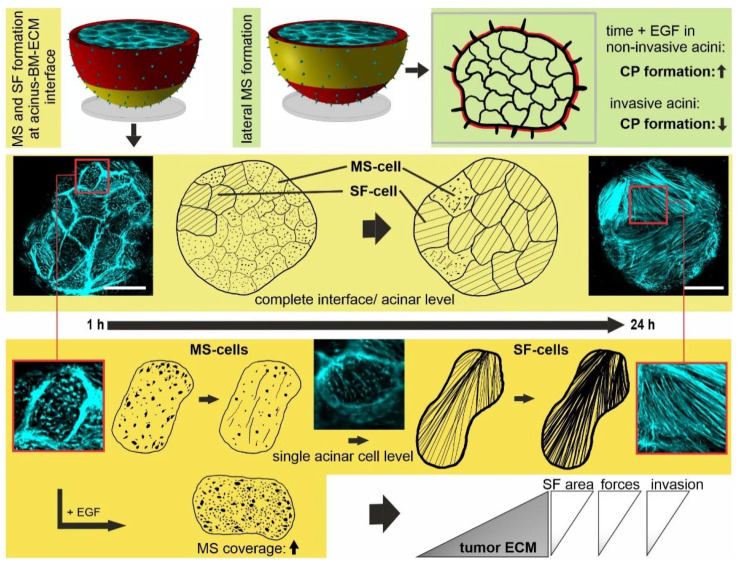
Graphical summary. A mechanistic model for the dynamic switch from non-proteolytic BM-traversing actin protrusions (CP) to highly contractile ventral stress fibers during the invasive transition of benign breast acini. Under non-invasive conditions and at the early stages of BM invasion, CPs act as mechanical probing units at the acinus–BM–ECM interface. In the course of the invasive transition, actin cytoskeleton remodeling mediates the switch from MS cells (rich in microspikes) to SF cells (rich in stress fibers). Oncogenic EGF and tumor-like ECM stiffness fuel this process. Progressively reinforced SF cells generate local hot spots of high contractile forces at the BM–ECM interface. This stress weakens the BM barrier chronically. The invasive transition of initially benign breast cells converges into BM disruption and finally cell invasion into the microenvironment.

The present study uncovered the inherent role of actin-rich CPs for the invasive transition of BM-covered benign breast acini (Figure 8). BM-piercing CPs functionally bridge the mechanical ECM-probing and the cell force-driven BM invasion mechanism. A tumorous microenvironment with deregulated EGF signaling and tumor-like stiffness synergistically reinforce stress fiber formation and, as a consequence, leads to high actomyosin II-driven cell forces. Finally, these EMT-like actin cytoskeletal adaptations fuel BM disruption in benign human breast cell acini. Since our cell model lacks a physiological 3-D matrix, future work should translate our findings into a cell model that allows investigating heterotypic cell–cell interactions, such as tumor cells and cancer-associated fibroblasts, within a three-dimensional ECM. Understanding mechanobiological mechanisms of cell adaptation to pathologic extracellular conditions offers high potential for developing efficient therapy approaches.

## Data Availability

The datasets supporting the conclusions of this article are either available within the paper and its supplementary information files or from the corresponding author upon reasonable request.

## Supplementary Materials

The following are available online at https://www.mdpi.com/article/10.3390/cells10081979/s1, Figure S1: The radii of breast acini depend on maturation state and EGF treatment, Figure S2: Invadopodia marker protein localization in single migrating cells and MCF10A acini, Figure S3: Focal adhesion and pMLCK localization in migrating MCF10-A cells, Figure S4: Traction force microscopy and hot spot segmentation, Movie S1: Lateral MS formation (Example 1), Movie S2: Lateral MS formation (Example 2), Movie S3: Lateral MS formation (Example 3), Movie S4: Migrating MCF10A cells, Movie S5: Movement of MCF10A acini on a tumor-like substrate (12 kPa), Movie S6: Stress fiber formation at the cell–BM–ECM interface (Example 1), Movie S7: Stress fiber formation at the cell-BM-ECM interface (Example 2).

## Abbreviations

BM	Basement membrane
CI	confidence interval
CP	cell protrusion
ECM	extracellular matrix
EGF	epidermal growth factor
FA	focal adhesion
FC	fold change
HD	hemidesmosomes
ld/hd-BM	low/highly-developed basement membrane
MMP	matrix metalloprotease
MS	microspike
pMLCK	phosphorylated myosin light chain kinase
SF	stress fiber
TFM	traction force microscopy
