# Supplementary material for: From Microspikes to Stress Fibers: Actin Remodeling in Breast Acini Drives Myosin II-Mediated Basement Membrane Invasion
Source: Cells. 2021 Aug 4;10(8):1979. doi: 10.3390/cells10081979 (PMC8394122; doi:10.3390/cells10081979)
Supplement: Supplementary file 1 [file cells-10-01979-s001.zip › cells-1301189-supplementary.pdf]

cells

Supplementary Information

From Microspikes to Stress Fibers: Actin Remodeling in Breast Acini Drives Myosin II-Mediated Basement Membrane Invasion.

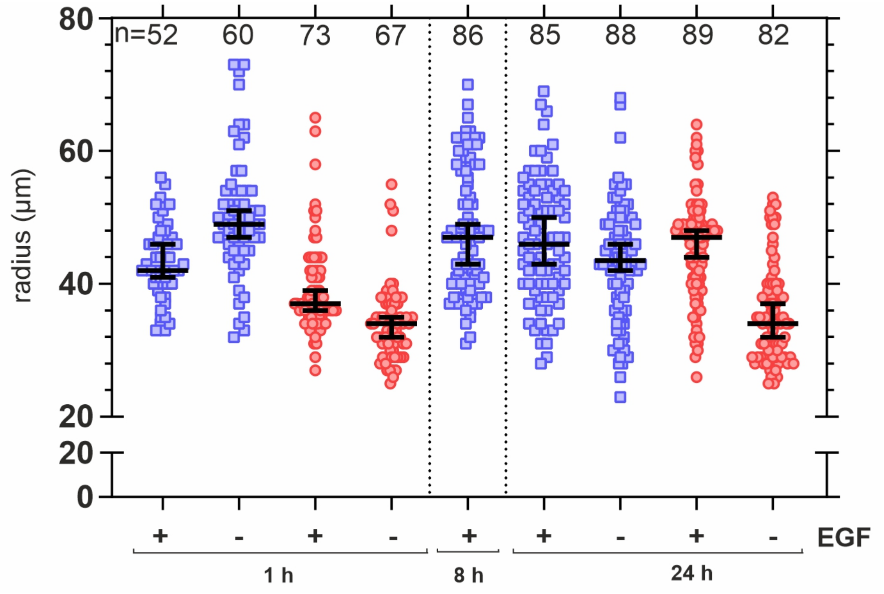

Figure S1. The radii of breast acini depend on maturation state and EGF treatment. Equatorial radii for BM perimeter calculation of all acini analyzed for Figures 2B and F. Maturation stages: 11 days old ld-BM acini (blue) and 21 days old hd-BM acini (red) with and without EGF treatment (20 ng/ml). Acini were transferred on a glass substrate and fixed after one, eight and 24 hours cultivation time with assay medium, either with EGF or without.

Cells **2021**, *10*, 1979. https://doi.org/10.3390/cells10081979

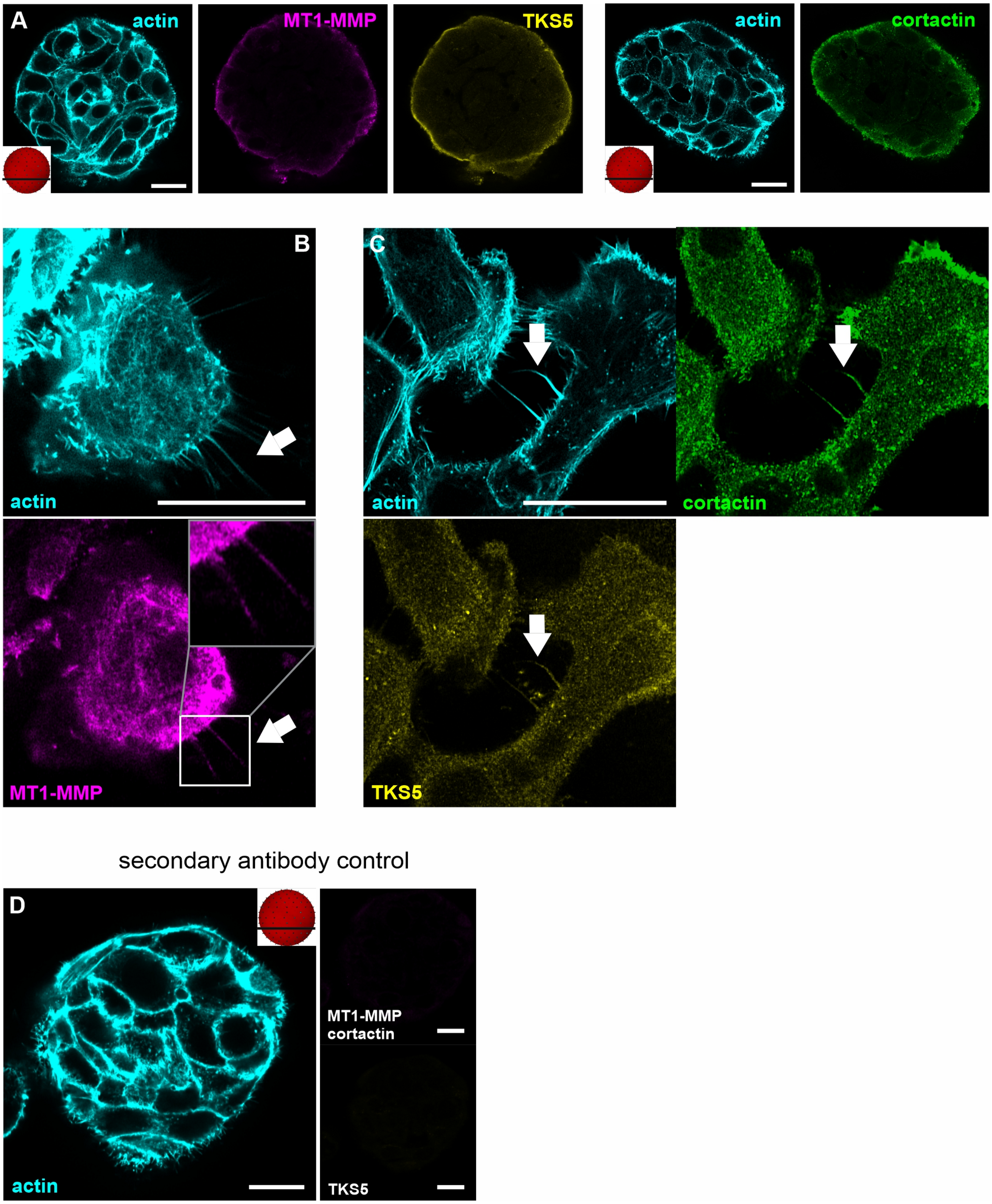

Figure S2. Invadopodia marker protein localization in single migrating cells and MCF10A acini. Confocal images of fixed ld-BM MCF10A acini sections or migrating cells on a planar glass substrate under most invasive conditions (24 h EGF treatment on a glass substrate). (**A**) The actin cortex (cyan) of the basal cell layer co-localizes with the invadopodia marker proteins MT1-MMP (magenta), Tks5 (yellow) and cortactin (green). For merged images see Figure 3A and B. (**B** and **C**) Single MCF10A cells on a planar substrate showing elongated actin-based filopodia-like cell protrusions positive for

MT1-MMP, TKS5 and cortactin. (**D**) Secondary antibody control for invadopodia staining shows no unspecific signals of secondary antibodies (cf. Figure 3Aand B). Scale bars = 20 µm.

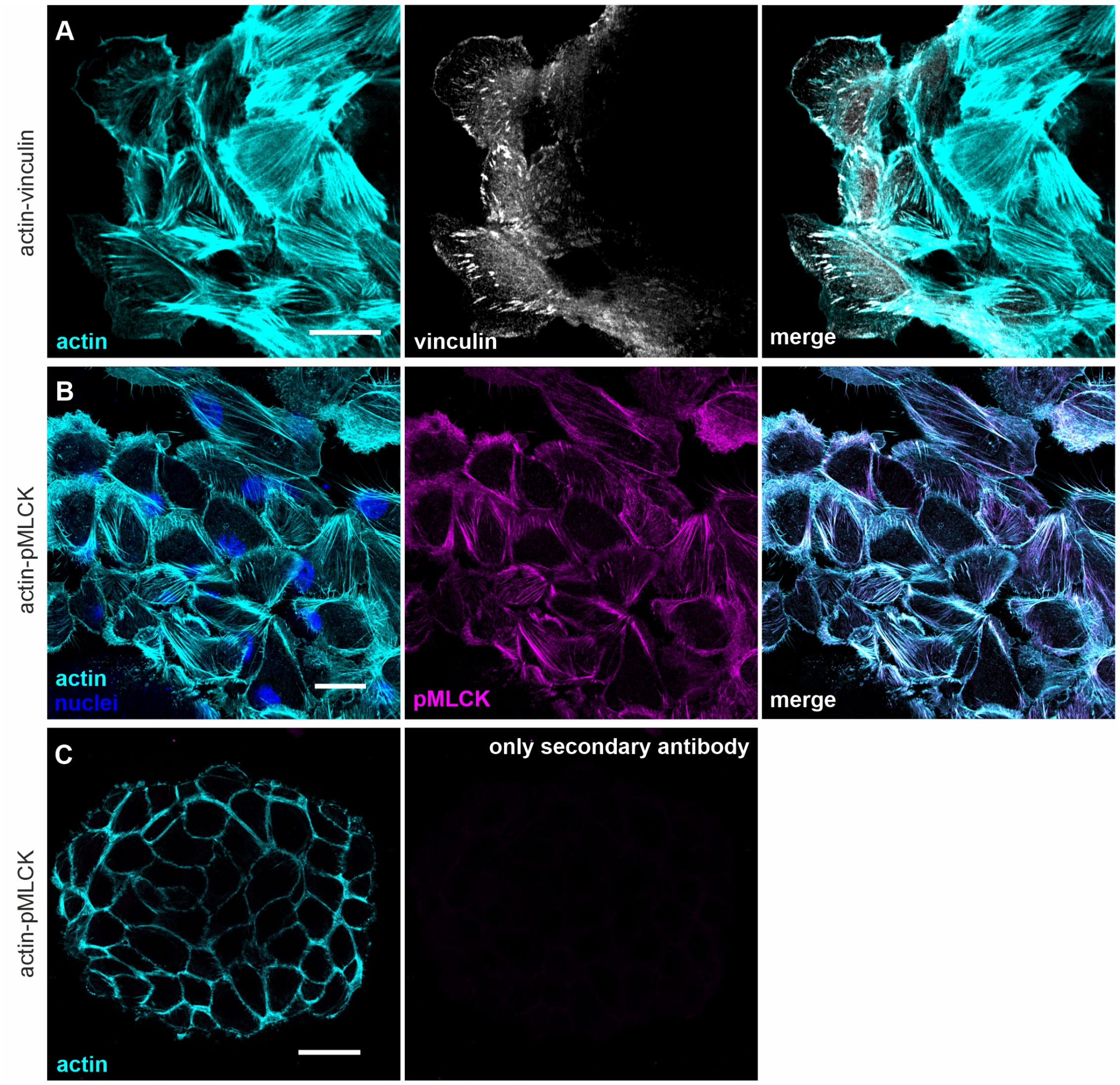

Figure S3. Focal adhesion and pMLCK localization in migrating MCF10-A cells. (**A**) Focal adhesions (vinculin) at ends of actin stress fibers in invasive MCF10A cells that left the acinar cell body. Cells are migrating on a planar glass substrate. (**B**) Representative images of MCF10A cells on a planar glass surface stained for phosphorylated myosin light chain kinase (pMLCK) and co-localization with the actin cytoskeleton. (**C**) Secondary antibody control for the pMLCK staining, showing no unspecific signal of the secondary antibody (cf. Figure 6G – H). Scale bars = 20 µm.

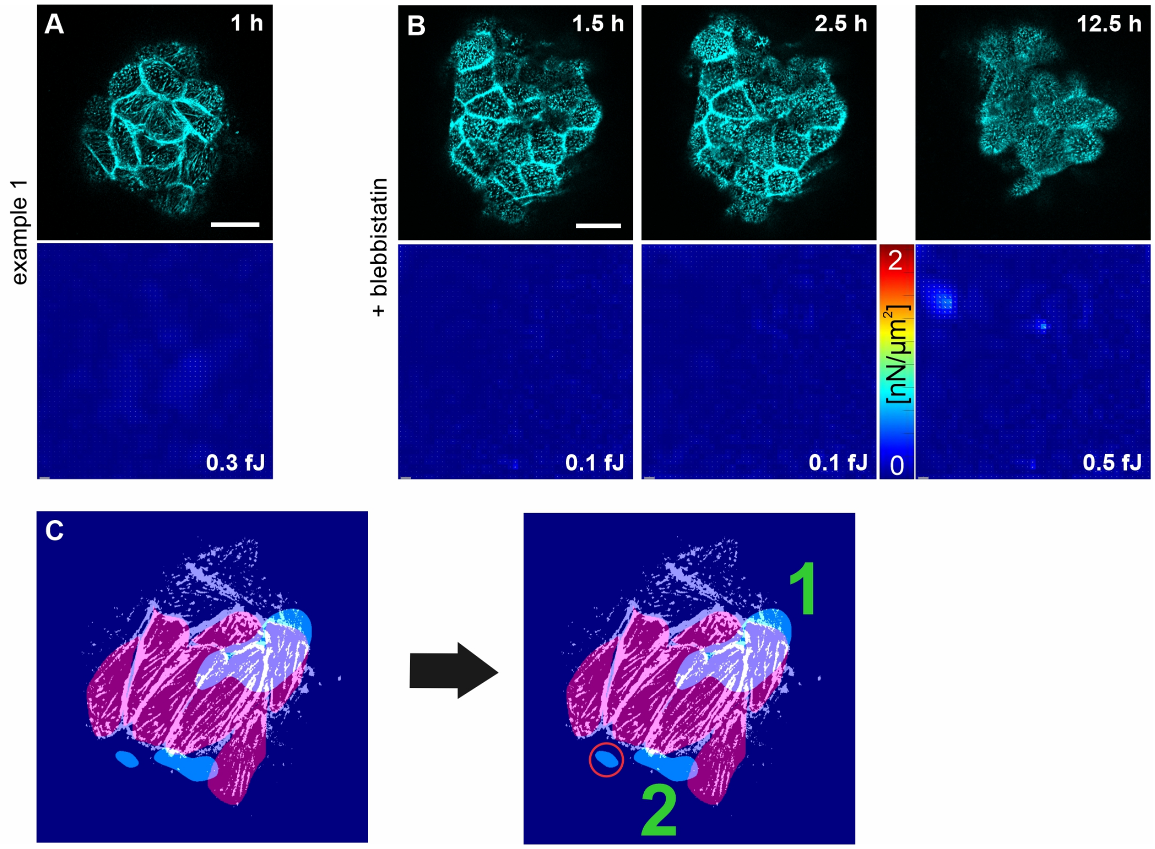

Figure S4. Traction force microscopy and hot spot segmentation. Shown are confocal images representing the actin cytoskeleton of basal cells at the BM-ECM interface and the corresponding substrate deformation-related stress fields (A and B). (**A**) The first image of Example 1 of Figure 7A (after 1 h) with sparse stress fiber formation, low substrate deformation, and accordingly low strain energies. (**B**) Further time points of ld-BM acini treated with blebbistatin (25 µM) (cf. Figure 7H). (**C**) Exemplary images illustrate two green numbered hot spots (in light blue; co-localized/covered with a magenta-colored SF-cell). Hot spots (red circle) localized at a distance to the actual acinus contact area were excluded from the analysis. Scale bar = 20 µm.

Movie S1: Lateral MS formation (Example 1). Illustration of the formation of a lateral MS by using a living MCF10A/RFP-LifeAct acinus. Confocal images were taken every 65 seconds for 18 minutes. Scale bar = 10 µm.

Movie S2: Lateral MS formation (Example 2). Illustration of the formation of lateral MS by using a living MCF10A/RFP-LifeAct acinus. Confocal images were taken every 55 seconds for 2 hours. Scale bar = 10 µm.

Movie S3: Lateral MS formation (Example 3). Illustration of the formation of lateral MS by using a living MCF10A/RFP-LifeAct acinus. Confocal images were taken every 55 seconds for 36 minutes. Scale bar = 10 µm.

Movie S4: Migrating MCF10A Cells. Visualization of living MCF10A/RFP-LifeAct cells migrating onto glass including the formation of filopodia or filopodia-like structures. Confocal images were taken every 31 seconds for 7 hours. Scale bar = 10 µm.

Movie S5: Movement of MCF10A acini on a tumor-like substrate (12 kPa). Illustration of cell movement of MCF10A wild-type cells within the acinar structure. Transmitted light images were taken every 20 minutes for 95 hours. Scale bar = 100 µm.

Movie S6: Stress fiber formation at the cell-BM-ECM interface (Example 1). Confocal images of a LifeAct/RFP MCF10A acinus. Scheme 1. depicted in Figure 7A. Images were taken every 7 minutes for 20 hours.

Movie S7: Stress fiber formation at the cell-BM-ECM interface (Example 2). Confocal images of a LifeAct/RFP MCF10A acinus. Shown is the complete actin cytoskeleton sequence of Example 2 depicted in Figure 7B. Images were taken every 7 minutes for 20 hours.
